# Effectiveness of educational intervention on women’s participation to cervical cancer screening: a quasi-experimental study based on PEN-3 model

**DOI:** 10.1186/s12885-022-10331-x

**Published:** 2022-11-28

**Authors:** Niloofar Seyrafi, Atefeh Homayuni, Zahra Hosseini, Teamur Aghamolaei, Amin Ghanbarnejad, Ali Mouseli

**Affiliations:** 1 Faculty of Health, Hormozgan University of Medical Sciences, Bandar Abbas, Iran; 2 Social Determinants in Health Promotion Research Center, Hormozgan Health Institute, Hormozgan University of Medical Sciences, Bandar Abbas, Iran

**Keywords:** Pap smear test, Cervical cancer, Intervention, PEN-3 model

## Abstract

**Introduction:**

Cervical cancer is one of the most common cancer types among women in developing countries. Women’s behavior in the early detection of the disease is influenced by sociocultural factors. Thus, the present study aimed to determine the effect of an educational intervention based on PEN-3 model on women’s participation in cervical cancer screening.

**Methods:**

The present quasi-experimental study was conducted with 160 women participants visiting health care centers in Bandar Abbas in 2021. The sampling was as a multi-stage clustering, and the participants were divided into two groups, an intervention and a control (each with 80 participants). The data collection instrument was a researcher-made questionnaire based on the PEN-3 model constructs before and 3 months after an educational intervention (a training course). The intervention involved 30 online sessions of 15–20 minutes for the intervention group while the control group did not receive any training.

**Results:**

After the educational intervention, the mean scores of knowledge, attitude, enablers, nurturers and the Pap smear test behavior in the experimental group increased significantly compared to the control group (*P* < 0.05). The analysis of covariance results showed that by modulating the effect of pre-test score, there was a statistically significant difference between the intervention and control groups in the post-intervention behavior score.

**Conclusion:**

In the light of the present findings, it can be concluded that interventions based on the PEN-3 model with a focus on knowledge -raising, changing beliefs and identifying sociocultural and environmental factors that affect cervical cancer screening behavior can prevent cervical cancer in women.

**Supplementary Information:**

The online version contains supplementary material available at 10.1186/s12885-022-10331-x.

## Introduction

Cervical cancer is a type of cancer that occurs in the cervix, the lower part of the uterus where the uterus joins the vagina. At the onset of the disease, there are usually no symptoms. Subsequent symptoms of the disease include vaginal bleeding, pelvic pain or pain during intercourse, and so on [1]. Cervical cancer is the fourth most malignant cancer that prevails among women worldwide and a major global health threat [2]. More than half a million women are diagnosed with cervical cancer every year, and the disease accounts for more than 300,000 mortalities on a global scale [3]. An early detection and prevention of cancer is essential in controlling the disease and increasing patient survival [4]. Because there is 10–20 years delay between the pre-cancerous stage and cervical cancer, there is a whale of time to diagnose and treat the cancer earlier and avoid its progression (pre-invasive period). Also, the availability of an appropriate screening program and effective treatment of primary lesions will contribute to the early diagnosis and prevention of death or disability [4, 5]. The Pap test is an effective screening program to this aim [2]. According to the National Cervical Cancer Program in Iran, screening should be done for all women after marriage, so women under 21 should have a Pap smear test after 3 years of their first intercourse and women over 21 after 1 year of their first intercourse [6]. Unfortunately,the existing facts and figures are not promising [5, 7, 8], for an instance, 49.9% of women had taken this test in their lifetime [9], while the rate in other countries was reported to be 85–93% [10].

Despite advances in screening and treatment over the past few decades, cervical cancer remains one of the major health issues among Iranian women. Research shows that women face many cultural, emotional, functional, social, religious, geographical and economic barriers to receiving services for the Pap smear test. Among the reasons for not adhering to this test are lack of knowledge about the need for Pap smear, lack of doctor’s advice, stress, embarrassment caused by the test, high cost, busy work, lack of privacy, fear of the positive test result, aggressiveness of the follow-up procedures (if the test turns positive) [11–13].

There are different models in health education and promotion to design, implement and evaluate preventive health programs, but none of them consider the culture of preventive behavior or health-promoting behavior at their core. Health beliefs and behaviors should be considered in cultural context [14, 15].

Health is an adaptive process and a cultural reaction to the reality of society. Citing culture as the most important factor in health education and health promotion programs requires a complete understanding of the cultural and political background of individuals’ health beliefs and behaviors. One health education and promotion model that focuses on culture in preventive behavior or health promotion at its core is the PEN-3 model. It includes three dimensions comprising the acronym PEN. These three dimensions are internally dependent on each other. The first dimension of the PEN-3 model is Health Education, which includes Person, Extended Family and Neighborhood. The second dimension is Educational Diagnosis which includes Perception, Enablers and Nurturers. The third dimension of the cultural fit model of health behaviors is Culture Adaptation, which means that cultural fit plays a central and essential role in planning health promotion and education interventions. This dimension views culture as a dynamic condition and interaction that is manifested in the behavior of the individual, family and society, and includes Positive, Existential and Negative aspects [14].

In Iran, few interventional studies have been conducted based on the PEN-3 model [11, 16]. Bandar Abbas is the capital city of Hormozgan Province and is one of the most important ports in the south of the country, located on the Persian Gulf and has different cultural and social factors due to the presence of different people from all over the country who live there.

Considering that the adoption of cervical cancer preventive behaviors is influenced by cultural issues [17], this study was conducted with the aim of determining the effect of educational intervention based on the PEN-3 model on women’s participation in cervical cancer screening.

## Methods

### Research design and population

The present study was a quasi-experimental intervention (with an intervention and a control group). The research population consisted of all women visiting healthcare centers in Bandar Abbas.

### Sample size and sampling procedure

In a similar study [18], the standard deviation of the behavior score (Pap smear) was equal to 1.5. with a standard error of 5%, an effect size of 0.7 and a power of 80%, the sample size was estimated using the following formula:

$$\textrm{n}=\frac{2{\left({\textrm{Z}}_{1-{}^{\upalpha }\!\left/ \!{}_{2\ }\right.}+{\textrm{Z}}_{1-\upbeta}\right)}^2\ {\textrm{s}}_{\textrm{p}}^2\ }{{\textrm{d}}^2}=144$$

To compensate for the attrition rate, 10% was added to the sample size, and finally the sample size was estimated at 160.

The sampling was done as multi-stage. In other words, in the first stage, the overall 20 comprehensive healthcare centers in Bandar Abbas were divided into two geographical regions (north and south). Each geographical region included 10 comprehensive health care centers. In the second stage, two health care centers were selected randomly from each region (in total, four comprehensive healthcare centers). In the third stage, two centers (one center from north and one from south) were randomly selected and assigned to the intervention group and two centers to the control group. Finally, in each center, the sample was selected from the active members whose records were kept in the SIB system of the centers. If they met the inclusion criteria, they were selected through convenience sampling method until the required sample size was met.

*Inclusion criteria:* age over 18 years, having an active record in the SIB system, a history of sexual intercourse and no history of Pap smear or no regular Pap smear recommended by the Ministry of Health, those over 21 years with more than 1 year passed since the last Pap smear, women under 21 years being married for at least 3 years, no history of cervical cancer, and consent to participate in the study, physical and mental health to participate in the study. *Exclusion criteria:* The exclusion criterion was failure to meet any one of the inclusion criteria.

### Data collection

The data collection phase coincided with the beginning of the Covid-19 pandemic in Iran. To assure the participants’ health, the educational intervention and data collection were done both in an online mode. The hyperlink to the questionnaire was created and sent to all participants in WhatsApp, and after a day, the participants were contacted to announce the completion of the questionnaire. A total number of 39 people had completed the questionnaire face to face. The rest of the questionnaires were completed on phone when there were cases of ambiguity. At the same time (on phone calls) the answers were recorded in the online survey (by the researcher). The average time was 18 minutes to complete the online survey by the individuals themselves and 33 minutes if done on phone and then transferred to the online survey. Before the intervention, the questionnaires were sent to participants in both intervention and control groups. Pre-test results were used to analyze the need for educational content, teaching methods and the number of sessions required for the training. Three months after the end of the training intervention, the data were collected again in both control and intervention groups**.**

### Instrumentation

We used researcher-made questionnaires to collect data. A panel of experts was formed to validate the questionnaire. The questionnaire was sent to health education and health promotion professors and a gynecologist, whose comments were used to enrich the questionnaire content. To determine the external reliability, the researcher conducted an experimental study with an interval of 10 days on 30 women (similar to the target research population) with a Test-Retest method. The internal consistency of the questionnaire was estimated using Cronbach’s alpha, which was estimated at 0.831 and the ICC was 0.89.

The questionnaires were: demographic information questionnaire, knowledge questionnaire and another questionnaire representing the PEN-3 constructs (see Supplementary file).

The demographic variables included: age, marital status, number of childbirths, age of first sex intercourse, level of education, occupation, and income. The second part of the questionnaire included 14 questions. A *Yes* answer received score 1 and a *No* or *Don’t know* answer received 0. The whole score ranged between 0 and 14, which assessed women’s knowledge of cervical cancer and the Pap test in Iran [18].

The PEN-3 questionnaire included 16 attitude questions, 12 enablers questions, 11 nurturers questions and 1 behavior question. Attitude questions enquired about the effectiveness of the Pap smear test, and that it helped diagnose and treatment of any lesion and malignancy in time. It explored attitude towards the effectiveness of the Pap test in reducing cervical cancer and the mortality rate. The questions were to be rated on a Likert scale ranging from strongly agree to strongly disagree, to be scored between 1 and 5. The minimum score was 12 and the maximum score was 80.

The questions about enablers explored the extent to which the respondent had access to the health center to receive information and services related to the test. It asked about the extent to which the health staff (in the health center and doctor’s office) were sufficiently skilled in the examination and conduction of the Pap test. The questions were rated on a Likert scale ranging from very high to very low and scored from 1 to 5. The minimum score was 12 and the maximum was 60.

Nurturers questions explored the extent to which the respondent’s husband objected to her presence at the health care center or doctor’s office for a Pap test. It also asked the extent to which the respondent’s doctor emphasized and recommended a Pap test. These were also rated on a Likert scale ranging from very high to very low, and rated from 1 to 5. The minimum score was 11 and the maximum was 55. The only behavior question “Have you ever done the pap test?” was to be answered as *Yes* (1 point) or *No* (0 point). A *Yes* answer was interpreted as showing the behavior and the *No* answer as the lack of behavior. In all three mentioned dimensions (attitude, enablers and nurturers), a higher score means a better and more positive attitude, more enablers (e.g., access to medical centers, health insurance coverage, etc.) and more nurturers (e.g., health staff and doctors, social media, etc.).

### Educational intervention

Due to the critical conditions caused by the Covid-19 pandemic, the curriculum was changed from face-to-face classes to e-learning.

We designed the intervention based on the effects on the model constructs and adopting the healthy behavior. The content of the training sessions was prepared from reliable sources. For each enabler and nurturer, we identified the environmental and cultural barriers based on the related literature and the briefing we had with the participants and provided the appropriate educational content.

Participants in the intervention group joined a WhatsApp group with 80 members. Educational interventions included training sessions for participants and family members or relatives. Thus, those present in the intervention group were asked to share the material with their husband, family members, friends and relatives after the training. The training program was held in a 4-month period and in 30 sessions of 15–20 minutes.

A group was formed in WhatsApp for the women in the intervention group. For a month, the daily educational content related to each of the model constructs was posted in the group. Concerning knowledge, during seven sessions, the materials covered were concerned about the anatomy of the uterus and cervix, cervical cancer disease, the status of cervical cancer in the world and Iran, cervical cancer symptoms, cervical cancer risk factors, ways to diagnose cervical cancer, screening and diagnosis. Also, the training involved personal hygiene and how to select the right contraceptive. The attitude was assessed during eight sessions. For two sessions, the people in the intervention group were asked to share their opinions and beliefs about cervical cancer, the Pap test and personal hygiene with the researcher. After that, for six sessions, each session addressed the prioritization of the issues raised about mental beliefs, positive and negative consequences of showing the behavior of interest, the factors facilitating the behavior and the motivation to follow the influential people around. The enablers were examined in ten sessions. Subjects such as time management with a focus on decision-making skills and prioritizing tasks, delegation of authority, perfectionism, and procrastination were discussed. The midwifery expert addressed issues such as the effect of insurance cards on the costs of screening and visiting a doctor, as well as the cultural barriers (high therapeutic costs, visits by a male doctor, non-confidentiality of client’s information, etc.) for the early diagnosis of cervical cancer, and at the end of the session, there were Q&As. Nurtures were explored within five sessions. These included 4 sessions for health care providers, with the aim of empowering women and giving sufficient information to clients about genital diseases and the need for the Pap screening test, as well as communicating with clients through WhatsApp to inform them of the services provided. In another session, a number of pamphlets were prepared and the participants were asked to submit them to their relatives (spouses, friends and acquaintances) and their families to increase knowledge of the symptoms of cervical cancer and the significance of screening. Also, how to access the instructional notes and the educational content of the country’s universities of medical sciences, midwifery magazines, reliable websites and archives of TV channels regarding cervical cancer screening to increase access to reliable resources were provided to the participants in the intervention group.

The control group did not receive have any training or educational content. They were only contacted for doing the survey.

The details of the training program are presented in Table 1.

**Table 1:** Details of the training program

PEN-3 model constructions	Cultural barriers	Environmental barriers	Intervention
**Attitude**	1. Feeling embarrassed. 2. Having incorrect beliefs such as: affliction with the disease is related to the fate and cannot be prevented.	_	1. Presence of a midwifery expert in WhatsApp group. 2. Holding 1 session of survey 3. Emphasis on vaginal examination and Pap smear screening as a must along with the effectiveness of early diagnosis of the disease for treatment. (2 training sessions via sending video clips and texts) 4. Investigating the consequences of negligence and failure to perform examinations in the incidence of disease and treatment. (1 training session via sending video clips and pamphlets) 5. Description of vaginal examination and how to perform Pap smear test. (1 training session via sending video clips and pamphlets) 6. Ensuring that the place of examination is private without the presence of a third party. (1 training session via sending video clips) 7. Explaining the things that a person should do before the examination (such as hygiene, etc.), use of clean sheets during the examination to eliminate the sense of embarrassment. (2 training sessions via video clips and pamphlets)
**Enablers**	1-Values and beliefs causing not to go for a Pap smear screening test	1. Lack of sufficient skills of health care providers to do examinations and conduct the screening test 2.The high cost of the screening test 3. No health insurance coverage. 4. Crowded comprehensive health care centers. 5- Long distance from healthcare centers and laboratories for the sample testing. 6. Too much work and lack of time	1. Necessary explanations regarding the area covered by each comprehensive health service center and its subdivisions, which provide faster access. (1 training session via sending text files and sending images) 2. Ensuring the level of skills and literacy of health care providers. (2 training sessions via sending voice messages) 3. Description of payment for Pap smear and laboratory test costs. (1 training session via sending video clips) 4. Description of insurance booklets coverage of total expenses. (2 training sessions via sending text files) 5. Teach time management skills and division of tasks. (3 training sessions via sending text files and video clips) 6. 1 session for Q&As
**Nurturers**	1. Husband’s disapproval 2. Disapproval of the husband’s family 3. Disapproval of the participant’s family 4. Mass media	_	1. Providing educational content for husbands, family members and spouse’s family. (1 training session via sending pamphlets) 2. Educating and empowering people to access reliable sources about cervical cancer and Pap smear test. (4 training sessions via sending video clips, voice messages and text files) 3. Forming a WhatsApp group to provide educational intervention and membership of health care providers in this group and use their experiences.

### Data analysis

The data were analyzed in SPSS24. Chi-square test and independent-samples T-test were used to compare demographic variables and the model constructs in two groups before and after the intervention. Due to the normality of data, paired-samples T-test and independent-samples T-test were run to evaluate the effect of the intervention. Covariance analysis and logistic regression were used to test the effectiveness of model constructs. Significance level was set at *p* < 0.05.

### Ethical considerations

This study received the ethical code of IR.HUMS.REC.1399.007 from the ethics committee of Hormozgan University of Medical Sciences. Prior to the study, participants were informed of the method and purpose of study, and were assured that all information collected would be kept confidential. Written consent was obtained from the women to participate in the study.

## Results

As the analyses revealed, the mean and standard deviation of participants’ age in the intervention and control groups were 32.36 ± 6.337 and 29.39 ± 4.40, respectively. The highest frequency in the control group was 46, which belonged to the 18–30 age group, and 45 in the intervention group which belonged to the 31–40 age group. These participants mostly held diplomas. Among them, 137 were housewives.

The data in Table 2 showed that before the intervention, there was no statistically significant difference between the two groups in terms of age, marital status, age of first sexual intercourse, number of childbirths, level of education, occupation, and socioeconomic status.

**Table 2:** Distribution of demographic variables in research groups

variables	Control group (***n***=80)	Intervention group (***n***=80)	***p***-value
Age (year)	f (%)	f (%)	
30–18	46 (57.7)	28 (35.3)	0.186
40–31	32 (40.1)	45 (56.6)	
50–41	2 (2.5)	6 (7.7)	
60–51	0 (0)	1 (1.3)	
marital status	f (%)	f (%)	
Single	0 (0)	5 (6.3)	0.944
Married	76 (95)	72 (90)	
divorced	3 (3.8)	3 (3.8)	
Widow	1 (1.3)	0 (0)	
Age of first sexual intercourse	f (%)	f (%)	
15–10	0 (0)	3 (3.8)	0.077
20–16	43 (53.8)	54 (67.7)	
25–21	34 (42.7)	17 (21.3)	
30–26	3 (3.8)	5 (6.3)	
35–31	0 (0)	1 (1.3)	
deliveries	f (%)	f (%)	
No childbirth	4 (5)	9 (11.3)	0.942
1	37 (46.3)	18 (22.5)	
2	28 (35)	36 (45)	
3	7 (8.8)	13 (16.3)	
4	2 (2.5)	4 (5)	
5	2 (2.5)	0 (0)	
Educational level	f (%)	f (%)	
Illiterate	1 (1.3)	0 (0)	0.807
Primary	2 (2.5)	3 (3.8)	
junior high school	6 (7.5)	16 (20)	
Diploma	43 (53.8)	40 (50)	
Associate Degree	9 (11.3)	6 (7.5)	
Bachelor	17 (21.3)	11 (11.8)	
Master’s degree and higher	2 (2.5)	4 (5)	
occupation	f (%)	f (%)	
Housekeeper	71 (88.8)	66 (82.5)	0.059
Employed	7 (8.8)	10 (12.5)	
Retired	0 (0)	1 (1.3)	
collegian	2 (2.5)	3 (3.8)	
Financial situation	f (%)	f (%)	
Weak	11 (13.8)	14 (17.5)	0.776
Medium	57 (71.3)	60 (75)	
Good	12 (15)	6 (7.5)	
Excellent	0 (0)	0 (0)	

The results related to the investigation of the relationship between age and age of first sexual intercourse with behavior are shown in Table 3. The results indicated that there was a significant difference between the mean age of participants with Yes and No responses (*p* < 0.0001) and according to the mean ranks, the mean age of participants with No response is higher than the mean age of participants with Yes response. In other words, younger participants are more likely to perform cervical cancer screening. The results also showed that there was no significant difference between the mean age of the first sexual intercourse of participants with YES and NO responses (*p* > 0.05).

**Table 3:** Results of Mann–Whitney U test of the demographic variables according to the behavior

Demographic variables	Age			Age of first sexual intercourse		
	N	Mean Rank	Sum of Rank	N	Mean Rank	Sum of Rank
Yes	28	48.32	1353	28	74.70	2091.50
No	132	87.33	11,527	132	81.73	10,788.50
Total	160			160		
Mann–Whitney U	947			1685.50		
Z	−4.055			−0.738		
Sig	0.000			0.461		

The results related to the examining the relationships between marital status, occupation status, deliveries and educational level with behavior are shown in Table 4. The results of the table indicated that there was a linear correlation of 0.254 between the two variables of marital status and behavior (*p* < 0.05). However, there were no linear correlation between the variables of occupation status, deliveries and educational level with the behavior (*p* > 0.05) and there was independence between these variables.

**Table 4:** Phi test results to investigate the relationship between demographic variables and behavior

Demographic variables	Behavior	
	**Value**	sig
Marital status	0.254	0.016
Occupation	0.113	0.566
Deliveries	0.000	1.000
Educational level	0.036	0.316

The results of Table 5 indicated that before the intervention, there was no statistically significant difference between the two groups (i.e., intervention and control) in knowledge, attitude, nurturers and behavior (*p* > 0.05). However, after the intervention, a statistically significant difference was observed between the two groups in all variables (p<0.05). In the intervention group, the mean behavior score increased from 0.662 to 1, which was statistically significant (*p* < 0.001).

**Table 5:** Comparison of mean scores, before and after the intervention in the research groups

Variable	Groups	Pre-test (Mean ± SD)	Post-test (Mean ± SD)	***P***-value
**Knowledge**	Intervention	8.5 ± 2.19	13.98 ± 0.111	<0.001 0.045
	Control	8.37 ± 2.06	8.57 ± 2.12	
	*P*-value	0.711	<0.001	
**Attitude**	Intervention	61.95 ± 3.78	63.16 ± 2.98	0.028 <0.001
	Control	62.66 ± 4.18	54.15 ± 7.37	
	*P*-value	0.261	<0.001	
**Enabling factors**	Intervention	39.55 ± 4.7	43.52 ± 3.94	<0.001 <0.001
	Control	41.3 ± 4.53	33.3 ± 5.13	
	*P*-value	0.018	<0.001	
**Nurturers**	Intervention	28.51 ± 2.89	33.5 ± 2.56	<0.001 <0.001
	Control	29.35 ± 3.06	32.46 ± 2.7	
	*P*-value	0.077	0.014	
		N (%)	N (%)	
**Behavior**	Intervention	54 (67.5%)	79 (98.8%)	< 0.001^*^ 0.125^*^
	Control	48 (60.0%)	52 (65.0%)	
	*P*-value	0.324	< 0.001	

In order to compare the mean post-test scores of the variables after controlling for the effect of pretest, an analysis of covariance was run and the results are summarized in Table 6.

**Table 6:** Analysis of covariance results of the mean scores in pre-test and post-test in the research groups

Variable	Source	Sum of squares	df	Mean square	F	***P***-value
**Knowledge**	group	1146.59	1	1146.59	842.55	<0.001
**Attitude**	group	3262.13	1	3262.13	102.67	<0.001
**Enablers**	group	3505 54	1	3505.54	7197.76	<0.001
**Nurturers**	group	3.3	1	3.3	0.472	493.0
**Behavior**	group	4.25	1	4.25	65.4	<0.001

Table 6 showed that after eliminating the effect of pre-test on dependent variables and according to the estimated F-values, the adjusted mean scores of knowledge (f = 842.55, *p* < 0.001), attitude (f = 102.67, *p* < 0.001), enablers (f = 197.769, *p* < 0.001), and behavior (f = 65.4, *p* < 0.001), revealed significant differences between the two groups in the post-test. The findings showed the effect of the intervention on the mentioned variables in the participants of the intervention group. The results summarized in the table also show a significant difference between the adjusted mean scores of nurturers (f = 0.472, *p* = 0.493) of the two groups in the post-test. Therefore, it can be concluded that the educational intervention did not have any effect on any of these variables.

The results of Table 7 showed that attitude and enabling factors with a significance level greater than 0.05 should be removed from the model. According to the significance level of knowledge and nurturers, which is less than 0.05, these variables should be present in the model.

**Table 7:** Logistic regression coefficient of PEN-3 model structures

Variable	(B)	Standard Error	Exp(B)	wald	df	*p*-value
Knowledge	3.752	1.491	42.599	6.335	1	0.012
Attitude	−0.005	0.071	0.995	0.005	1	0.945
Enabling factors	0.015	0.061	1.015	0.057	1	0.811
Nurturers	−0.304	0.062	0.709	31.192	1	0.001

## Discussion

The present study investigated the effect of an educational intervention based on PEN-3 model on women’s behavior of cervical cancer screening. The results showed that the mean scores of knowledge increased after the intervention, and a statistically significant difference was found between the two groups in terms of the knowledge before and after the intervention (p<0.001). This finding is similar to a body of research conducted by Pirzadeh and Amidi Mazaheri [19], Adamu et al. [20], Gana et al. [21], Karimy et al. [22], Williams et al. [23] and Jeihooni et al. [24] and Jeihooni et al. [25]. The findings reported by Pirzadeh and Amidi Mazaheri [19] also confirm that education increased women’s knowledge of the Pap smear test in the intervention group. The findings reported by Adamu et al. [20] on the effect of health education on knowledge, attitude and practice of Pap smear test among female teachers showed the mean knowledge score of cervical cancer was low in both intervention and control groups. After the intervention, a significant increase was observed in the knowledge score of the intervention group. The results of the study by Jeihooni et al. [24] in evaluating the effect of educational intervention on the promotion of prostate cancer screening in a sample of Iranian men showed health education programs based on the PRECEDE model improved knowledge, attitude, enablers, perceived social support, quality of life, overall health and self-efficacy. The program showed to have a positive effect on prostate cancer screening behaviors.

We also found that the mean attitude score in the intervention group had a significant increase after the intervention compared to the control. Because attitude is a predisposing factor for the onset and persistence of healthy behaviors and is correlated with behavior, a positive attitude can be a strength in promoting screening behavior [15, 16, 18]. A significant increase in attitude in the intervention group is consistent with a body of research by Adamu et al. [20], Mirzaei-Alavijeh [19] and Jeihooni et al. [24]. The research findings by Adamu et al. [20] showed that the mean score of attitude towards Pap smear test was low in both intervention and control groups, but after the intervention the attitude score was significantly increased in the intervention group. In other words, the percentage of participants with a positive attitude towards the Pap smear test was significantly higher in the intervention group than the control. Among the reasons reported for participants’ low performance was their reluctance to perform a Pap smear and the belief that the test was redundant. Iranian women showed that after the intervention, a significant improvement was achieved in the positive attitude towards Pap smear test.

Findings about enablers showed that the mean score had a significant increase in the intervention group in the post-test compared to the control (p<0.001). Enablers are considered to be the prerequisite for behavioral change, and can be motivating. This finding was consistent with the results of studies conducted by Williams et al. [23], Jeihooni et al. [24], Dizagi et al. [26] and inconsistent with the results of the study conducted by Abedi et al. [27]. Williams et al. [23] examined the socio-cultural and structural factors associated with cervical cancer screening among HIV-infected African Americans in Alabama. They found that among the most common positive attitude, enablers and nurturers that contributed to cervical cancer screening were intrinsic motivation and the perceived importance of Pap smear testing for HIV-infected women due to their weakened immune systems. Negative perceptions, negative enablers and nurturers included a lack of knowledge about cervical cancer and screening, and the perceived lack of susceptibility to cervical cancer. Dizaji et al. [26] explored the effect of an educational intervention based on the Precede model on self-care and control behaviors in patients with type II diabetes. These researchers found that the mean scores of knowledge, attitude, practice, enablers and nurturers increased after the educational intervention. Abedi et al. [27] explored the effect of education on promoting self-care behaviors in patients with hypertension based on the Precede model. Their study showed that the educational intervention did not increase the score of enablers in the intervention group. One reason for the contradictory findings is the time of study which began at the same time as the outset of the National Blood Pressure Control Plan by the Ministry of Health in the country and, therefore, at the same time as the intervention group, the control group also received training and related services through the media [19].

We also found that the mean scores of nurturers in both intervention and control groups were significantly higher in the post-test than the pretest. Nurturers can lead to continued behavior and give ongoing rewards for maintaining behavior. This finding is consistent with a body of research by Williams et al. [23], Jeihooni et al. and Abedi et al. [24, 27], yet inconsistent with the results of the study by Mirtz et al. [28]. The effect of social support and the encouragement by family and important others to motivate preventive behaviors such as cervical cancer screening was an important finding by Williams et al. [23]. Jeihooni et al. [24] investigated Iranian men and found no significant difference between the social support mean scores of the control and intervention groups before training. However, after the training intervention, the between-group differences became significant. Mirtz et al. [28] focused on screening idiopathic scoliosis to improve the educational, social and clinical health performance of adolescents using the Prescott-Process model. These researchers showed that the educational intervention did not increase the nurturer score in the intervention group. Among the reasons for this discrepancy was the difference in sample size and research population, the measurement instrument and the type of behavior investigated. Chen pointed out that social support promotes self-care behavior and influences participation and self-care behavior through social interaction [29]. Recording the information of the participants (in both the control and intervention groups) in the form of a questionnaire (pre-test), led to the curiosity and inquiry of the participants from the officials of the health care centers, and these officials are obliged to guide and inform the participants. This led to informing women about the services provided by health care centers, which they may have been unaware of it. In addition, participants in control group may have received encouragement from their family, friends, peers, health workers, community leaders and mass media during this time about participation in screening. Therefore, the scores of nurturers in post-test were increased in both groups compared to pretest.

Finally, the results showed that the mean behavior score of the intervention group in the post-test was significantly higher than the control. This finding was in line with the results of the studies conducted by Jeihooni et al. [24], Pirzadeh and Amidi Mazaheri [19], Romli et al. [30], Daryani et al. [31], yet inconsistent with the results of the study conducted by Adamu et al. [20]. In another investigation conducted by Pirzadeh and Amidi Mazaheri [19], 97.14% of the participants in the intervention group performed the Pap smear test after the intervention. However, only 2.86% of the control group did so. Romli et al. [30] recruited female entrepreneurs in the northern state of Malaysia and found that the Pap smear test in the intervention group changed significantly from 48% (in the baseline state) to 68% (in evaluation 1) and from 68 to 79% in the next evaluation. A significant increase was also observed in the Pap smear test of the control group from 63% (in the baseline state) to 76% in the first stage of evaluation. Daryani et al. [31] conducted their study to determine the effectiveness of an educational intervention based on the health belief model in women’s practice of the Pap smear test. They showed that, after the intervention, there was a significant difference between the two groups in terms of the practice of the Pap smear test. However, the results reported by Adamu et al. [20] showed that the proportion of women’s practice of Pap smear test was low in both groups, in the baseline and after the intervention and also between the two stages in the intervention group. Respondents were asked why they did not take the test. Prior to the intervention, 52.3% of the intervention group and 63.6% of the control group reported that they were not asked to do so. 18.2% of the intervention group and 5.2% of the control group felt they did not need a Pap smear test. 6.7% of the intervention group and 1.3% of the control group stated that they did not like to do the test.

Considering the positive effects of the educational intervention based on the PEN-3 model, it is suggested to use this model in other areas of health promotion and health behavior in women. It is also recommended to plan for designing and implementing educational interventions based on the PEN-3 model to reduce cultural and environmental barriers to making healthy decisions in other populations of the society.

To conduct this study, the method of sampling among visitors to health care centers was convenience in type. During the Covid-19 pandemic, people visited healthcare centers significantly less than before. Thus, our sampling was restricted. We suggest that in future studies, the researchers use random sampling from among the list of households to make it possible to include more samples (greater sample size and more diverse population).

### Limitations

There are certain limitations in the present study including the small sample size and, thus, the limited generalizability of findings to the whole population, failure to hold face-to-face training in the Covid-19 pandemic and the limited communication with people. Due to the coincidence of the study with the prevalence of coronavirus in the world and the risk of transmission of coronavirus through holding face-to-face training and for protecting the health of participants, we hold intervention sessions online. Due to the cultural and environmental barriers in our country (Table 1), the presence of women in medical centers make them familiar with treatment environment and women’s diseases, as well as the services they could receive and online sessions created restrictions in this regard. One strength of the study is that it was pioneering in adopting the PEN-3 model to prevent cervical cancer in Iran and Bandar Abbas.

## Conclusion

The present study indicated the effectiveness of the PEN-3 model in increasing cervical cancer screening behavior (Pap smear) and clinical examination. According to the PEN-3 model, at first, the perceptual, cultural (e.g., embarrassment and unavoidable cancer) and environmental factors (e.g., lack of access to centers and laboratories, the high cost of screening) affecting behavior were identified. Then, an appropriate intervention program was designed and implemented based on the PEN-3 model. Attempts were made to increase the participants’ knowledge, attitude, enablers, nurturers and screening behavior. The increase in Pap smear screening behavior and clinical examination in the intervention group (after the intervention) indicates the effectiveness of this intervention at the community level to reduce the incidence of the later stages of cancer through regular cervical cancer screening procedures. Therefore, interventions based on the PEN-3 model with an emphasis on raising knowledge, changing beliefs and identifying socio-cultural and environmental factors affecting cervical cancer screening behavior are highly recommended.

## Supplementary Information

**Additional file 1.** Questionnaire.

## Data Availability

The datasets generated and analyzed during the current study are not publicly available due to confidentiality and privacy related issues but are available from the corresponding author on reasonable request.

## References

[1] Bray F, Laversanne M, Weiderpass E, Soerjomataram I (2021). The ever-increasing importance of cancer as a leading cause of premature death worldwide. Cancer.

[2] Bedell SL, Goldstein LS, Goldstein AR, Goldstein AT (2020). Cervical cancer screening: past, present, and future. Sex Med Rev.

[3] Cohen PA, Jhingran A, Oaknin A, Denny L (2019). Cervical cancer. Lancet.

[4] Hossaini F, Tahmasebi R, Noroozi A (2017). Comparing the effects of individual and group training methods based on health belief model on the belief and practice of Bushehr women regarding pap smear test. J Health.

[5] Khiyali Z, Asadi R, Ghasemi A, Khani JA (2017). The effect of educational intervention base on health belief model on performing pap smears in women of Fasa city. Iran J Health Educ Health Promot.

[6] Eghbal SB, Karimy M, Kasmaei P, Roshan ZA, Valipour R, Attari SM (2020). Evaluating the effect of an educational program on increasing cervical cancer screening behavior among rural women in Guilan, Iran. BMC Women's Health.

[7] Riahi S, Mokhtari AM, Vali M, Abdzadeh E, Mohseni S, Salehiniya H (2019). Incidence and mortality rate of cervix cancer in Iran from 1990 to 2016: a systematic review and meta-analysis. J Contemp Med Sci.

[8] Ghahramani S, Kasraei H, Shahabi S, Lankarani KB. Facilitating factors and barriers of women's cancer screening in Iran: a systematic review. International J Prev Med. 2020:11;199. 10.4103/ijpvm.IJPVM_509_18 PMC8000176 33815723

[9] Shabgard BMM, Khajehvand A. The effect of educational intervention based on BASNEF model on life style of Cancer in female in Rasht. Med J Mashhad Univ Med Sci. 2020;63(2):2499–507.

[10] Nazari S, Keshavarz Z, Afrakhte M, Riazi H (2019). Barriers to cervical Cancer screening in HIV positive women: a systematic review of recent studies in the world. Iran J Epidemiol.

[11] Parsa P, Aghababaii S, Rahmani S (2020). Cervical cancer screening in postmenopausal women referring to Hamadan comprehensive health centers. Pajouhan Sci J.

[12] Hajializadeh K, Ahadi H, Jomehri F, Rahgozar M (2014). The role of health Beliefsin predicting Barriersto cervical Cancer screening. J Kerman Univ Med Sci.

[13] Lemp JM, De Neve J-W, Bussmann H, Chen S, Manne-Goehler J, Theilmann M (2020). Lifetime prevalence of cervical cancer screening in 55 low-and middle-income countries. JAMA.

[14] Shahbazi H, Ghofranipour F, Amiri P, Rajab A. Factors affecting self-care performance in adolescents with type i diabetes according to the PEN-3 cultural model. Int J Endocrinol Metab. 2018;16(4):e62582. 10.5812/ijem.62582 PMC6216475 30464772

[15] Blackstone SR, Nwaozuru U, Iwelunmor J (2018). Antenatal HIV testing in sub-Saharan Africa during the implementation of the millennium development goals: a systematic review using the PEN-3 cultural model. Int Quarterly Commun Health Educ.

[16] Naghibi A, Jamshidi P, Yazdani J, Rostami F (2016). Identification of factors associated with breast cancer screening based on the PEN-3 model among female school teachers in Kermanshah. Iran J Health Educ Health Promot.

[17] Xia X, Jiang W, Qi W, Hong B, Zhao W (2020). Clinical efficacy and safety of Apatinib for the treatment of patients with metastatic, recurrent cervical Cancer after failure of radiotherapy and first-line chemotherapy: a prospective study. Oncol Res Treat.

[18] Naghibi A, Rostami F, Masomi F, Cherati JY (2016). Application of the perceptual factors, enabling and reinforcing model on pap Smaear screening in Iranian northern woman.

[19] Pirzadeh A, Mazaheri MA (2012). The effect of education on women's practice based on the health belief model about pap smear test. Int J Prev Med.

[20] Adamu A, Abiola A, Ibrahim M (2012). The effect of health education on the knowledge, attitude, and uptake of free pap smear among female teachers in Birnin-Kebbi, North-Western Nigeria. Niger J Clin Pract.

[21] Gana GJ, Oche MO, Ango JT, Kaoje AU, Awosan KJ, Raji IA. Educational intervention on knowledge of cervical cancer and uptake of Pap smear test among market women in Niger State, Nigeria. J Public Health Africa. 2017;8(2):575. 10.4081/jphia.2017.575 PMC5793045 29416840

[22] Karimy M, Gallali M, Niknami S, Aminshokravi F, Tavafian S (2012). The effect of health education program based on health belief model on the performance of pap smear test among women referring to health care centers in Zarandieh. J Jahrom Univ Med Sci.

[23] Williams M, Moneyham L, Kempf M, Chamot E, Scarinci I (2015). Structural and sociocultural factors associated with cervical cancer screening among HIV-infected African American women in Alabama. AIDS Patient Care STDs.

[24] Jeihooni AK, Kashfi SM, Hatami M, Avand A, Bazrafshan M (2019). The effect of educational program based on PRECEDE model in promoting prostate cancer screening in a sample of Iranian men. J Cancer Educ.

[25] Khani Jeihooni A, Jormand H, Harsini PA (2021). The effect of educational program based on beliefs, subjective norms and perceived behavior control on doing pap-smear test in sample of Iranian women. BMC Womens Health.

[26] Dizaji MB, Taghdisi MH, Solhi M, Hoseini SM, Shafieyan Z, Qorbani M (2014). Effects of educational intervention based on PRECEDE model on self care behaviors and control in patients with type 2 diabetes in 2012. J Diabetes Metab Disord.

[27] Abedi M, Borhani M, Rahimzadeh H, Mehri A, Hoseini ZS (2020). The role of education in promoting self-care behaviors in patients with hypertension: an application of the PRECEDE model. J Educ Commun Health.

[28] Mirtz TA, Thompson MA, Greene L, Wyatt LA, Akagi CG (2005). Adolescent idiopathic scoliosis screening for school, community, and clinical health promotion practice utilizing the PRECEDE-PROCEED model. Chiropractic Osteopathy.

[29] Chen S-Y, Wang H-H (2007). The relationship between physical function, knowledge of disease, social support and self-care behavior in patients with rheumatoid arthritis. J Nurs Res.

[30] Romli R, Sa’adiah Shahabudin NS, Mokhtar N. (2020). Effectiveness of a health education program to improve knowledge and attitude towards cervical cancer and pap smear: a controlled community trial in Malaysia. Asian Pac J Cancer Prev.

[31] Daryani S, Shojaeezadeh D, Batebi A, Charati JY, Naghibi A (2016). The effect of education based on a health belief model in women's practice with regard to the pap smear test. J Cancer Policy.

